# Real-world incidence and risk factors of bortezomib-related cardiovascular adverse events in patients with multiple myeloma

**DOI:** 10.1007/s44313-024-00004-y

**Published:** 2024-02-19

**Authors:** Bitna Jang, Jonghyun Jeong, Kyu-Nam Heo, Youngil Koh, Ju‑Yeun Lee

**Affiliations:** 1 College of Pharmacy and Research Institute of Pharmaceutical Sciences, Seoul National University, 1, Gwanak-Ro, Gwanak-Gu, Seoul, 08826 Republic of Korea; 2 Department of Pharmacy, Seoul National University Hospital, Seoul, Republic of Korea; 3 Department of Internal Medicine, Seoul National University Hospital, Daehak-Ro Jongno-Gu, 101 Seoul, Republic of Korea

**Keywords:** Bortezomib, Proteasome inhibitors, Adverse drug events, Hypertension, Cardiotoxicity

## Abstract

**Background:**

Although most studies on the cardiovascular toxicity of proteasome inhibitors have focused on carfilzomib, the risk of cardiotoxicity associated with bortezomib remains controversial. This study aimed to evaluate the incidence and risk factors of cardiovascular adverse events (CVAEs) associated with bortezomib in patients with multiple myeloma in a real-world setting.

**Methods:**

This cross-sectional study included patients who were treated with bortezomib at a tertiary hospital in South Korea. CVAEs, defined as hypertension, arrhythmia, heart failure, myocardial infarction, pulmonary arterial hypertension, angina, and venous thromboembolism, were detected using cardiac markers, ECG, echocardiography, medications, or documentation by clinicians. The patients were observed for at least 6 months and up to 2 years after starting bortezomib administration.

**Results:**

Among the 395 patients, 20.8% experienced CVAEs of any grade, and 14.7% experienced severe adverse events. The median onset time for any CVAE was 101.5 days (IQR, 42–182 days), and new-onset/worsened hypertension was the most prevalent CVAE. The risk of CVAEs increased in patients with a body mass index lower than 18.5 (adjusted HR (aHR) 3.50, 95% confidence interval (CI) 1.05-11.72), light chain (1.80, 1.04-3.13), and IgD (4.63, 1.06-20.20) as the multiple myeloma subtype, baseline stroke (4.52, 1.59-12.80), and hypertension (1.99, 1.23-3.23). However, CVAEs did not significantly affect the 2-year overall survival and progression-free survival.

**Conclusion:**

Approximately 15% of the Korean patients treated with bortezomib experienced severe CVAEs. Thus, patients, especially those with identified risk factors, should be closely monitored for CVAE symptoms during bortezomib treatment.

**Supplementary Information:**

The online version contains supplementary material available at 10.1007/s44313-024-00004-y.

## Introduction

Multiple myeloma (MM) is a type of blood cancer characterized by malignant plasma cell neoplasms in the bone marrow that lead to bone destruction and suppression [1]. It is the third most common type of blood cancer after leukemia and non-Hodgkin’s lymphoma. Over the past two decades, the number of patients with MM has increased by approximately 30 times [2]. Traditionally, alkylating agents and anthracyclines have been used to treat MM. However, newer drugs, such as immunomodulators and proteasome inhibitors, have been approved, resulting in changes in treatment and improved survival rates over the last 15 years [3–6].

The ubiquitin–proteasome pathway is essential for regulating the degradation of unnecessary or damaged proteins in cells [7]. In MM cells, abnormal ubiquitin–proteasome system regulation leads to proteasome overactivation, causing p53 and NF-KB inhibitor degradation. As a result, MM cells suppress apoptosis, promote angiogenesis and proliferation, and promote tumor progression [8]. Since the approval of bortezomib, the first proteasome inhibitor, in 2003, bortezomib has become the cornerstone of MM treatment [9].

Bortezomib, an anticancer drug that targets the 26 s proteasome, inhibits the ubiquitin–proteasome pathway. It was approved for the treatment of MM in South Korea in 2006 Clinical guidelines such as those of the National Comprehensive Cancer Network (NCCN), recommend bortezomib as the primary therapy for MM in combination with dexamethasone and immunomodulators [10]. In South Korea, bortezomib is the primary therapy for patients with MM and is covered by the National Health Insurance (NHI). Although carfilzomib-based combination therapy is recommended as the preferred regimen in the NCCN guidelines, the Korean NHI covers only carfilzomib after prior chemotherapy has failed.

Proteasomes play an important role in maintaining the heart size and structure by regulating the generation and decomposition of heart-building proteins. Proteasome inhibitors are known to cause cardiovascular complications such as heart failure, cardiomyopathy, stable angina, hypertension, and thromboembolism [7]. Among the proteasome inhibitors commonly used in clinical practice, carfilzomib, an irreversible proteasome inhibitor, is considered to be the most strongly associated with cardiovascular complications. The higher cardiotoxicity of carfilzomib has been suggested to be caused by its irreversible proteasome inhibition and its distinctive molecular mechanism, which includes AMPKα inactivation and autophagy-related protein downregulation [11]. In a network meta-analysis of clinical trials, the risk of cardiovascular toxicity was over 2.5 times higher in patients treated with carfilzomib than in those who received control treatment [12]. Most studies investigating the cardiovascular toxicity of proteasome inhibitors have focused on carfilzomib [13].

However, the risk of cardiotoxicity remains controversial, with the reported incidence varying considerably. A retrospective single-center study found that cardiotoxicity occurred in 14% of patients receiving bortezomib [14]. In a prospective study that assessed cardiovascular adverse events (CVAEs), including venous thromboembolism and hypertension, CVAEs occurred in 17% of the 30 patients who received bortezomib for relapsed MM. The authors also reported that CVAEs significantly reduced overall survival (OS) and progression-free survival (PFS) [15]. However, a meta-analysis of clinical trials reported a lower cardiotoxicity incidence of approximately 4% for bortezomib, which was not significantly different from that of controls [16]. Another study using a claims database in the US found that the risk of cardiotoxicity was not statistically higher in patients receiving bortezomib therapy than in those receiving lenalidomide [17].

Moreover, it is important to investigate the cardiotoxicity associated with proteasome inhibitors because of the increased risk of cardiovascular disease in patients with MM. The median age of patients with MM is 69 years, and 69% have preexisting cardiovascular comorbidities at the time of diagnosis [18, 19]. Immunoglobulin accumulation in MM can result in arrhythmia and heart failure [20].

The occurrence of CVAEs can significantly affect the clinical outcomes of patients with MM, highlighting the importance of identifying their incidence and related risk factors. Although a case report documented reversible heart failure following bortezomib treatment in a Korean patient with MM [21], the specific cardiotoxicities associated with bortezomib have yet to be thoroughly investigated. Data on the Korean population might yield different insights owing to variations in pharmacogenetic profiles compared to those of Western populations.

Given the insufficient evidence of CVAEs in patients treated with bortezomib, this study aimed to estimate the incidence of CVAEs, explore the risk factors, and assess their impact on clinical outcomes in Korean patients with MM receiving bortezomib treatment in real-world practice.

## Materials and methods

### Study participants

For this retrospective cross-sectional study, we included patients with MM aged 18 years or older who received chemotherapy, including bortezomib, at Seoul National University Hospital from February 1, 2007, to December 31, 2019. We excluded patients: (1) not diagnosed with MM; (2) with less than 6 months of follow-up; (3) diagnosed with light chain cardiac amyloidosis based on medical history, electrocardiogram, echocardiography, proteinuria, biopsy, or cardiac MRI; (4) who participated in a clinical trial; (5) Individuals who exhibited the following conditions or newly developed diseases within 3 months before commencing bortezomib treatment: symptomatic arrhythmia necessitating treatment, New York Heart Association (NYHA) class 3 or higher symptoms, myocardial infarction, pulmonary arterial hypertension, angina (cardiac chest pain), venous thromboembolism, or initiation of 1 or more cardiovascular medications.

The patients were observed for at least 6 months and up to 2 years after the start of bortezomib administration. We censored the patients at the time of death, transferred them to another hospital, and lost them to follow-up. We imputed missing values in the patient data using the mean of the remaining patients. Regarding the smoking status, patients were classified as nonsmokers in cases where smoking information was absent.

This study was approved by the Medical Research Ethics Review Committee of Seoul National University Hospital (IRB No. H-2001–132-1096). The need for informed consent was waived by the ethics committee due to the retrospective study design.

### Definition of outcomes

CVAEs included hypertension, arrhythmia, heart failure, myocardial infarction, venous thromboembolism, pulmonary arterial hypertension, and angina (cardiac chest pain). Each AE was graded based on the National Cancer Institute Common Terminology Criteria for Adverse Events (CTCAE) version 5.0 [22]. CVAEs were objectively determined using cardiac markers, ECG, echocardiography, medication use, or documentation by clinicians in medical records. Hypertension was defined as a systolic blood pressure of 160 mmHg or greater or diastolic blood pressure of 100 mmHg observed twice in a row or newly added antihypertensives, corresponding to CTCAE grade 3 or higher; therefore, grade 1/2 hypertension was not identified. Arrhythmia was detected by a physician after the electrocardiogram. Heart failure was diagnosed when a patient met at least 2 of the following criteria: documented heart failure symptoms, abnormalities on physical examination, and abnormal laboratory findings. New-onset venous thromboembolism was detected using a clinician’s document based on imaging records. Pulmonary arterial hypertension was detected using echocardiography and physical examination. Angina and cardiac chest pain were documented by the physician when new symptoms were observed and a clear causal relationship between bortezomib treatment and these symptoms was established (Supplementary Table S2). Causality was assessed according to the World Health Organization-Uppsala Monitoring Centre (WHO-UMC) causality assessment scale and the only events assessed as having possible or higher causality were included. CVAEs were observed for up to 56 d after bortezomib discontinuation or alternative anticancer therapy initiation, whichever occurred first.

To identify risk factors for bortezomib-related CVAEs, patient demographics (sex, age, body mass index [BMI], tobacco use, family history of cardiovascular diseases, myeloma subtype, chemotherapy regimen, MDRD-GFR, serum albumin, anemia, ≥ 1 prior chemotherapy, prior radiotherapy, prior autologous hematopoietic cell transplantation, baseline diseases [diabetes, stroke, angina, hypertension, dyslipidemia, arrhythmia, and heart failure]) were evaluated as candidate variables.

### Statistical analysis

Continuous variables were presented as means and standard deviations or medians and ranges. Categorical variables were presented as frequencies. We present cardiovascular adverse events as cumulative incidence. The CVAE risk factors were identified using a multivariable Cox proportional hazards model. The analysis results are presented as 95% confidence intervals and *P*-value. OS and PFS were evaluated and compared between the groups with and without CVAEs using the Kaplan–Meier method and log-rank test. Cumulative incidence and Kaplan–Meier plots were analyzed using the R Studio 1.4.1717 version software, and other analyses were performed using the SAS software (version 9.4; SAS Institute, Cary, NC, USA).

## Results

### Population characteristics

**Fig. 1:**
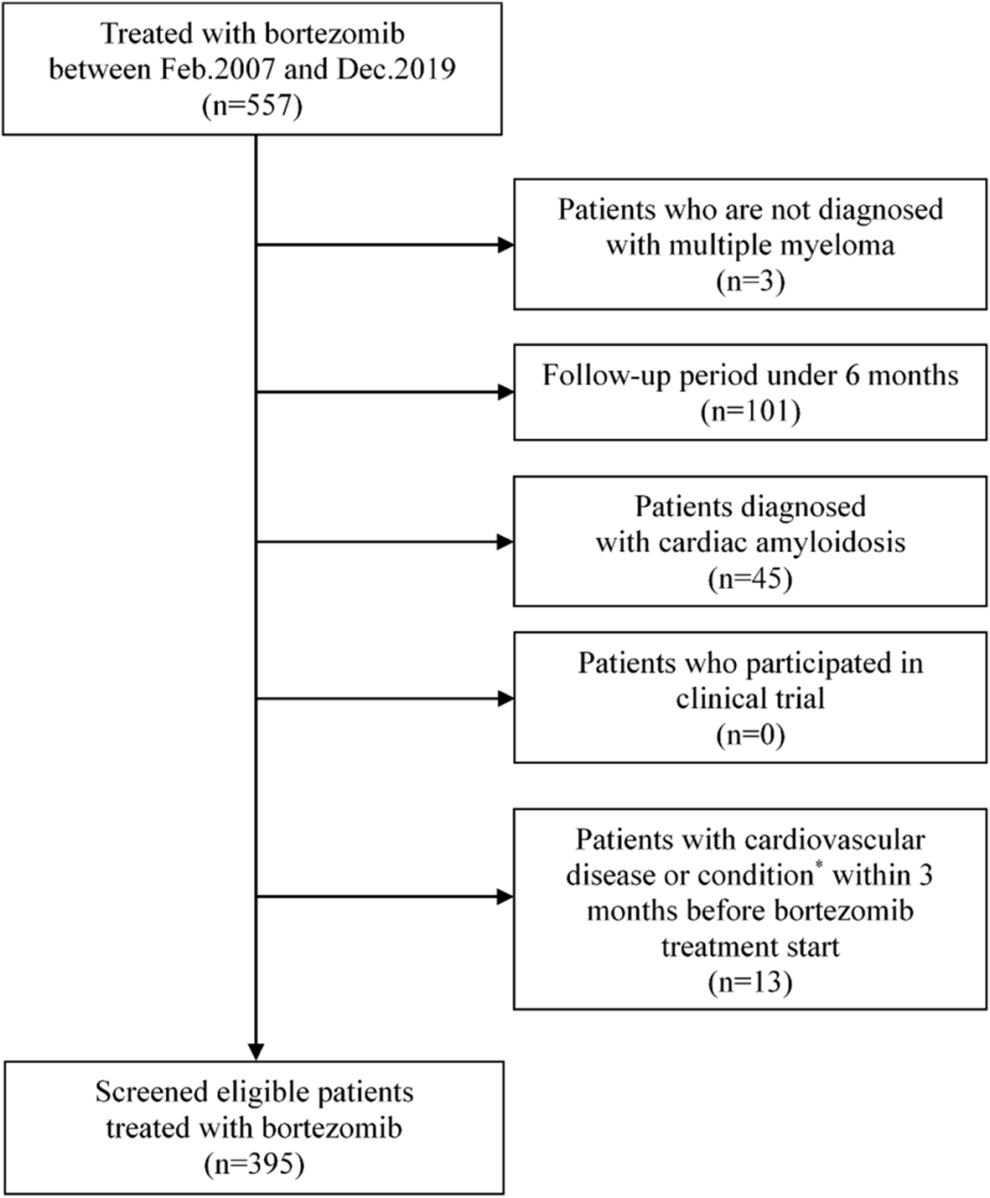
Selection process of patients with multiple myeloma treated with bortezomib included in the study

In total, 557 patients received bortezomib-based chemotherapy during the study period. After excluding patients without MM diagnosis (*n* = 3), with less than a 6-month follow-up period (*n* = 101), those with a disease/condition of interest at baseline (*n* = 13), and those diagnosed with cardiac amyloidosis (*n* = 45), 395 patients were included in the analysis (Fig. 1). The median follow-up period was 730 d (IQR, 658–730). The mean age of the patients was 67.6 years and 54.4% were men. The major MM subtype was IgG-type (51.4%), followed by light chain (22.5%) and IgA-type (15.7%). Approximately two-thirds (65.8%) of patients were chemotherapy-naïve. The bortezomib, melphalan, and dexamethasone triple combination, bortezomib-dexamethasone, and bortezomib-thalidomide-dexamethasone comprised 35.4%, 34.4%, and 28.1% of patients, respectively (Table 1).

**Table 1:** Baseline characteristics of patients treated with bortezomib (*N* = 395)

Characteristics	N (%)
Sex, Male	215 (54.4)
Age, years, mean ± SD	67.6 ± 9.9
< 65	137 (34.7)
≥ 65	258 (65.3)
Body mass index (BMI), kg/m^2^, mean ± SD	24.1 ± 3.2
< 18.5	254 (64.3)
≥ 18.5	141 (35.7)
Tobacco use	23 (5.8)
Family history of cardiovascular disease	65 (16.5)
Myeloma subtype	
Ig G	203 (51.4)
Ig A	62 (15.7)
Ig D	3 (0.8)
Light chain	89 (22.5)
Non-secretory	6 (1.5)
Not available	32 (8.1)
Baseline MDRD-GFR, ml/min/1.73m^2^, mean ± SD	70.1 ± 30.7
< 30	55 (13.9)
≥ 30	340 (86.1)
Baseline serum albumin, g/dL, mean ± SD	3.7 ± 0.6
< 3.5	305 (77.2)
≥ 3.5	90 (22.8)
Baseline hemoglobin, g/dL, mean ± SD	10.6 ± 2.1
13.0 g/dL for men, 12.0 g/dL for women	82 (20.8)
Prior lines of chemotherapy, median (IQR)	0 (0–4)
0	260 (65.8)
1	94 (23.8)
2	29 (7.3)
3 +	12 (3.0)
Prior radiotherapy	46 (11.6)
Prior autologous hematopoietic cell transplantation	76 (19.2)
Underlying diseases	
Hypertension	172 (43.5)
Diabetes	87 (22.0)
Arrhythmia	62 (15.7)
Dyslipidemia	53 (13.4)
Stable angina	22 (5.6)
Heart failure	19 (4.8)
Stroke	10 (2.5)
Chemotherapy regimens	
Bortezomib, melphalan, and dexamethasone	140 (35.4)
Bortezomib and dexamethasone	136 (34.4)
Bortezomib, thalidomide, and dexamethasone	111 (28.1)
Other bortezomib based regimen	8 (2.0)

*Ig* Immunoglobulin, *MDRD-GFR* Estimated glomerular filtration rate using the modification of diet in renal disease formula, *SD* standard deviation, *IQR* interquartile range

### Incidence and risk factors of bortezomib-related CVAEs

Among the 395 patients who received bortezomib, 82 (20.8%) and 58 (14.7%) experienced CVAEs of any grade and grade 3, respectively. Only one patient discontinued bortezomib because of a cardiovascular adverse event, which was identified as deep vein thrombosis.

Hypertension had the highest incidence rate (11.4%). The incidence of heart failure of any grade was 4.3%, and that of grade 3 or higher was 1.8%. Any grade of arrhythmia, myocardial infarction, angina, or venous thromboembolism occurred in 3.3%, 2.8%, 2.5%, 1.3%, and 0.8% of patients, respectively. The median onset time for any CVAE was 101.5 days (IQR, 42–182). Myocardial infarction of any grade demonstrated the earliest onset (median, 42 days; IQR, 14–95), and the median onset times of heart failure, hypertension, arrhythmia, pulmonary hypertension, angina, and venous thromboembolism were 91, 102, 114.5, 121, 123, and 138 days, respectively (Table 2).

**Table 2:** Incidence of bortezomib-related cardiotoxicity (*N* = 395)

	**Number of events (N)**	**Median onset time** **days (IQR)**	**Incidence (%)**
Any cardiovascular adverse events			
Any grade	82	101.5 (42–182)	20.8
Severe	58	101.5 (31–182)	14.7
Hypertension			
Any grade	45	102 (23–165)	11.4
Severe	45	102 (23–165)	11.4
Arrhythmia			
Any grade	10	114.5 (17–188)	2.5
Severe	2	99 (10–188)	0.5
Heart failure			
Any grade	17	91 (47–212)	4.3
Severe	7	174.5 (44.5–254.5)	1.8
Angina (cardiac chest pain)			
Any grade	13	123 (74–286)	3.3
Severe	3	372 (108–604)	0.8
Myocardial infarction			
Any grade	5	42 (14–95)	1.3
Severe	2	311 (95–527)	0.5
Pulmonary hypertension			
Any grade	11	121 (97–212)	2.8
Severe	2	326 (280–372)	0.5
Venous thromboembolism			
Any grade	3	138 (56–475)	0.8
Severe	1	56 (-)	0.3

*IQR* interquartile range

**Fig. 2:**
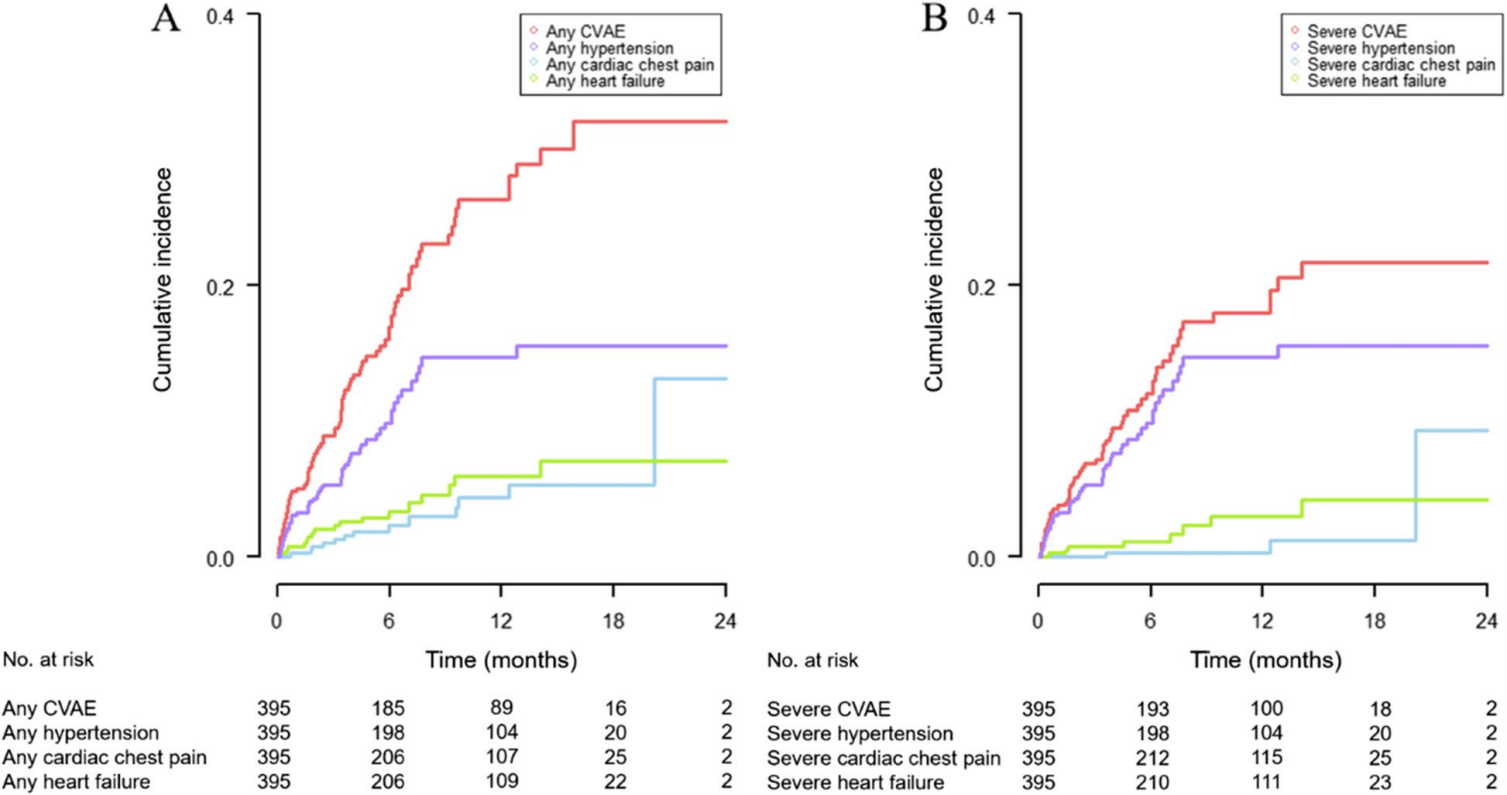
Cumulative incidence of any grade (**A**) and grade 3 or higher (**B**) bortezomib-related cardiovascular adverse events (CVAEs)

The cumulative incidence of any CVAE was 32.1% at 2 years, and that of grade 3 or higher CVAE was 21.7% at 2 years. The cumulative incidence of any grade of hypertension, angina, and heart failure were 15.5%, 13.1%, and 7.1% at 2 years, respectively (Fig. 2 and Supplementary Table S2).

Multivariable analysis demonstrated that history of stroke (HR 4.52, 95% CI 1.59–12.80), baseline hypertension (HR 1.99, 95% CI 1.23–3.23), Ig D type (HR 4.63, 95% CI 1.06–20.20), low BMI (HR 3.50, 95% CI 1.05–11.72), and light chain type (HR 1.80, 95% CI 1.04–3.13) increased the risk of CVAEs (Table 3).

**Table 3:** Univariable and multivariable analysis of risk factors for bortezomib-related cardiotoxicity

Characteristics	Univariable		Multivariable	
	HR	95% CI	aHR	95% CI
Sex				
Male	(Ref)	-	-	-
Female	1.41	0.91–2.20	-	-
Age				
< 65 years	(Ref)	-	-	-
≥ 65 years	0.82	0.51–1.33	-	-
Body mass index (kg/m^2^)				
< 18.5	2.51	0.78–8.05	3.50	1.05–11.72
18.5–25	(Ref)	-	(Ref)	-
≥ 25	0.92	0.58–1.46	0.91	0.56–1.47
Tobacco use				
Non-smoker	(Ref)	-	(Ref)	-
Smoker	2.24	1.12–4.47	2.09	1.00–4.37
Family history of cardiovascular diseases	1.22	0.69–2.13	-	-
Myeloma subtype				
IgG	(Ref)	-	(Ref)	-
IgA	0.79	0.38–1.63	0.63	0.30–1.33
IgD	3.37	0.81–13.97	4.63	1.06–20.20
Light chain	2.06	1.25–3.39	1.80	1.04–3.13
Non-secretory	1.27	0.17–9.25	1.52	0.20–11.65
Not available	0.90	0.35–2.28	0.84	0.32–2.21
MDRD-GFR, ml/min/1.73m^2^				
< 30	2.29	1.37–3.82	1.28	0.68–2.43
≥ 30	(Ref)	-	(Ref)	-
Albumin, g/dL				
< 3.5	1.44	0.90–2.32	-	-
≥ 3.5	(Ref)	-	-	-
Hemoglobin				
< 13.0 g/dL for men, < 12.0 g/dL for women	1.54	0.83–2.84	-	-
≥ 13.0 g/dL for men, ≥ 12.0 g/dL for women	(Ref)	-	-	-
Prior chemotherapy	0.42	0.24–0.73	0.61	0.29–1.25
Prior radiotherapy	0.90	0.45–1.80	1.62	0.77–3.43
Not received autologous hematopoietic cell transplantation	2.77	1.28–6.02	1.77	0.65–4.87
Underlying diseases				
Dyslipidemia	0.66	0.32–1.36	-	-
Diabetes	1.17	0.71–1.94	-	-
Stroke	4.85	1.94–12.11	4.52	1.59–12.80
Stable angina	1.07	0.43–2.64	-	-
Hypertension	2.14	1.37–3.34	1.99	1.23–3.23
Arrhythmia	1.74	1.04–2.90	-	-
Heart failure	1.41	0.61–3.24	-	-
Chemotherapy regimen				
Bortezomib and dexamethasone	(Ref)	-	-	-
Bortezomib, melphalan, and dexamethasone	1.50	0.85–2.66	-	-
Bortezomib, thalidomide, and dexamethasone	2.60	1.41–4.78	-	-
Other bortezomib based regimen	1.59	0.21–12.01	-	-

*MDRD-GFR* estimated glomerular filtration rate using the modification of diet in renal disease formula, *aHR* adjusted hazard ratio, *CI* confidence interval

### Impact of bortezomib-related CVAE on clinical outcome

**Fig. 3:**
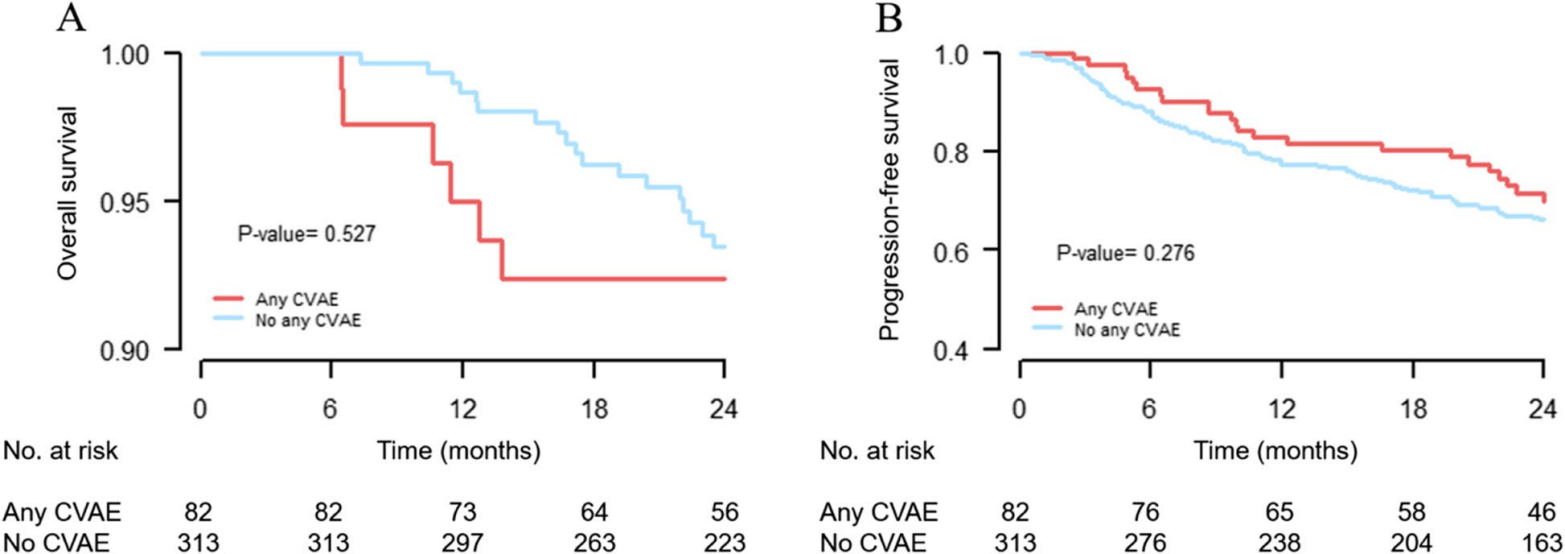
The overall survival (**A**) and progression-free survival (**B**) according to the occurrence of any cardiovascular adverse events (CVAEs)

At a median follow-up of 24 months (IQR, 21.9–24 months), the 2-year OS and PFS rates were 93.2% and 66.9%, respectively. The OS of patients who experienced CVAE (92.4% at 2 years) and those who did not experience any CVAEs (93.5% at 2 years) was not significantly different. In addition, the PFS of patients who experienced any CVAEs (69.9% at 2 years) was not significantly different from that of patients who did not (66.1% at 2 years) (Fig. 3).

## Discussion

This study demonstrated that the incidence of CVAEs during bortezomib-based chemotherapy in patients with MM was 20.8% for any grade and 14.7% for grade 3 or higher. The incidence of CVAEs observed in this study was higher than the 3.8% reported in a meta-analysis [16] and the 15% reported in a phase 3 clinical trial [23]. Differences in population, AE detection methods, and definitions of cardiovascular toxicities may explain the discrepancy in incidence. A meta-analysis of clinical trials defined cardiovascular outcomes as heart failure, cardiomyopathy, left ventricular ejection fraction decline, arrhythmia, and cardiac arrest. However, new-onset or worsening hypertension and thromboembolism were excluded. Additionally, clinical trial participants may have excluded those with baseline cardiovascular comorbidities [24].

The incidence of grade 3 hypertension (11.4%) contributed to the high CVAE incidence in this study. The observed hypertension incidence in this study was lower than that reported by 26% from a multicenter prospective observational study [15]. The definition of severe hypertension used in a previous prospective observational study was the same as that used in this study. However, we limited our study to two consecutive occasions, which may have contributed to a slightly lower incidence. The long-term use of high-dose steroids such as dexamethasone or prednisone in combination therapy can also cause fluid retention, contributing to hypertension [20].

In the present study, the severe heart failure and arrhythmia incidence rates were 1.8% and 0.5%, respectively. This was similar to a large retrospective analysis that reported a 1.2–4.7% incidence of severe heart failure and 0·6–4·1% of severe arrhythmia in relapsed or refractory MM [25]. However, the heart failure incidence was lower than 13.0% observed in a multicenter prospective observational study [15]. This discrepancy can be explained by the retrospective design of this study.

The median onset date of any grade of CVAEs after bortezomib was 101.5 days from the start of treatment, with a wide quartile range of 42–182 days. The median onset of myocardial infarction was the earliest at 42 days, whereas that of venous thromboembolism was the latest at 138 days. Unlike a recent study that mainly reported CVAEs within 3 months [16], this study demonstrated that CVAEs often occurred in the later phase of bortezomib treatment and that monitoring of CVAEs during this period may be necessary.

In the context of our study, the inclusion of patients with MM and pre-existing cardiovascular conditions was a deliberate choice aimed at reflecting the complex clinical profiles commonly encountered in real-world settings. This inclusion could potentially confound the assessment of bortezomib-related CVAEs. To mitigate this, we applied specific exclusion criteria, as detailed in the Methods section, targeting patients who exhibited significant cardiovascular conditions or developed new diseases within three months before initiating bortezomib treatment. This approach is essential for discerning the direct impact of bortezomib on cardiovascular health, separating it from the effects of pre-existing or newly developed cardiovascular conditions. Therefore, our findings offer insights into the cardiovascular safety of bortezomib in a realistic clinical context where pre-existing cardiovascular diseases are common among patients with MM.

We identified low BMI, light chain, and IgD as the MM type, and pre-existing stroke and hypertension as risk factors for CVAEs in patients on bortezomib. Obesity is a well-known risk factor for cardiovascular disease; however, it was not found to be a risk factor for CVAEs in our study. Although we could not explain this, a previous study by Park et al. [26] found that even underweight patients had a higher risk of developing cardiovascular diseases such as stroke and myocardial infarction. Further studies are required to confirm these findings. The light chain type as a risk factor for CVAEs confirmed the clinical case reports by the Mayo Clinic, which reported that the light chain type was associated with CVAEs [27]. Like light-chain myelomas, IgD is a rare type of MM. In our study, the IgD type was identified as a significant risk factor for CVAEs. However, this observation was based on a very limited sample size, encompassing only three patients with this specific subtype. This small sample size introduces a considerable margin of potential statistical variability and uncertainty to our findings. Additionally, our results contrast with those of existing research. Notably, studies on IgD-associated amyloidosis have suggested a lower frequency of cardiac involvement [28], which diverges from our findings, indicating an increased risk. This discrepancy underscores the need for a cautious and nuanced interpretation of our results. Given the rarity of IgD MM and conflicting evidence in the existing literature, our conclusions regarding the risk of CVAEs in this specific patient population should be considered preliminary. Further research involving larger cohorts and more diverse patient demographics is crucial to validate and understand the cardiovascular risks associated with IgD-associated myeloma. We identified pre-existing hypertension as a risk factor, indicating that patients who already have hypertension when starting bortezomib treatment are more likely to experience worsening hypertension and may require more intensive treatment or face other cardiovascular events. Hypertension is widely recognized as a risk factor in these cases [29]. Although pre-existing stroke has not been reported in other studies, this finding comes from the common risk factors for stroke and cardiovascular events defined in this study [30].

This study found that bortezomib-related CVAEs did not affect the OS or PFS, contradicting the results of a previous study [15]. An earlier study mainly focused on patients who experienced carfilzomib-related CVAEs and reported significantly inferior PFS and OS compared to those who did not. Our study population included patients with bortezomib-related CVAEs, and higher PFS and OS. These differences may partly explain why we did not observe a significant effect of CVAE on the OS or PFS in our study.

This real-world study, which included nearly 400 patients treated with bortezomib for MM, provided important insights into the incidence and risk factors associated with bortezomib-related CVAEs. As patients with cancer often have underlying medical conditions, our findings can assist clinicians in identifying and monitoring patients who may be more susceptible to CVAEs during bortezomib treatment.

Although this study provided valuable insights, it is important to acknowledge its limitations. First, the retrospective study design and reliance on electronic records may have led to an underestimation of the incidence of CVAEs. This is because physicians may not document all symptoms and patients may not notice or report some symptoms. To address this limitation, laboratory values were considered along with clinical judgment to define the occurrence of CVAEs. Furthermore, in our initial study design, orthostatic hypotension, despite its reported association with bortezomib treatment and links to autonomic neuropathy, dehydration, and antihypertensive use [31], was not included in the CVAE outcomes. This omission stems from challenges in accurately identifying such cases owing to incomplete documentation in routine clinical practice. Second, confounding variables, such as neuropathy and thrombocytopenia due to bortezomib that caused frequent dose adjustments and changes in dosing schedules or the effect of steroids in combination therapy, were not fully accounted for in the analysis. Third, our study did not fully explore the potential contribution of combined chemotherapeutic agents, such as melphalan and thalidomide, to CVAEs. Although CVAEs specifically associated with melphalan, an older chemotherapeutic agent, have not been extensively documented, melphalan-induced supraventricular tachycardia and ventricular arrhythmias [32, 33]. In contrast, thalidomide is notably associated with thromboembolic events [34]; however, other types of CVAEs are not significantly associated with its adverse effect profile. This highlights the necessity of considering the cumulative cardiovascular impact of these agents in conjunction with bortezomib in the risk assessment of our patient population. Our study primarily focused on bortezomib, without an in-depth evaluation of the cardiotoxic potential of melphalan and thalidomide, which is a limitation to our comprehensive understanding of the multifaceted nature of CVAEs in the treatment of MM.

Fourth, this study included CVAEs with possible causality assessments linked to bortezomib. However, we acknowledge the potential for the misattribution of CVAEs to other causes. The advanced age of most patients with MM, along with an increased risk of cardiovascular events due to aging, the presence of the disease itself, preexisting comorbidities, prolonged steroid use leading to fluid retention, and myeloma-related anemia, may all contribute to CVAE incidence. Consequently, determining the specific contribution of bortezomib to the incidence of CVAEs remains challenging.

Furthermore, the absence of a comparison group was a significant limitation. This absence restricted our ability to establish a definitive cause-and-effect relationship between bortezomib use and CVAE incidence. Future studies with control or comparison groups are necessary to provide a more comprehensive understanding of the relationship between bortezomib and CVAEs.

## Conclusions

In a real-world setting, approximately 21% and 15% of Korean patients experience any grade of CVAEs and severe CVAEs, respectively, during bortezomib treatment. Our study identified low BMI, light chain, and IgD as MM types and pre-existing stroke and hypertension as risk factors for developing bortezomib-related CVAEs. Therefore, patients with these risk factors should be closely monitored during bortezomib treatment.

### Supplementary Information

**Additional file 1:** **Table S1.** Modified grade definition of cardiovascular events of interest based on the CTCAE-based grading system. **Table S2.** Cumulative incidence of bortezomib-related cardiovascular adverse events.
