# Supplementary material for: Real-world incidence and risk factors of bortezomib-related cardiovascular adverse events in patients with multiple myeloma
Source: Blood Res. 2024 Feb 19;59(1):3. doi: 10.1007/s44313-024-00004-y (PMC10903519; doi:10.1007/s44313-024-00004-y)
Supplement: Supplementary file 1 — Additional file 1: Table S1. Modified grade definition of cardiovascular events of interest based on the CTCAE-based grading system. Table S2. Cumulative incidence of bortezomib-related cardiovascular adverse events. [file 44313_2024_4_MOESM1_ESM.docx]

**Supplementary table**

| **Table S1. Modified grade definition of cardiovascular events of interest based on the CTCAE-based grading system** | | | | |
| --- | --- | --- | --- | --- |
|  | **Grade 1** | **Grade 2** | **Grade 3** | **Grade 4** |
| Hypertension | Systolic BP 120–139 mm Hg or diastolic BP 80–89 mm Hg | \| Systolic BP 140–159 mm Hg or diastolic BP 90–99 mm Hg if previously WNL; change in baseline medical intervention indicated; recurrent or persistent (≥24 hours); symptomatic increase by >20 mm Hg (diastolic) or to >140/90 mm Hg; monotherapy indicated initiated \| \| --- \| | Systolic blood pressure ≥160 mmHg or diastolic BP ≥100 mmHg *on two consecutive measures**; medical intervention indicated; more than one drug or more intensive therapy than previously used indicated | Life-threatening consequences (e.g., malignant hypertension, transient or permanent neurologic deficit, hypertensive crisis); urgent intervention indicated |
| Arrhythmia | Asymptomatic; Intervention not indicated | Non-urgent medical intervention indicated | Urgent intervention indicated | Life-threatening consequences; hemodynamic compromise |
|  | *Evaluated by electrocardiogram and documentation by a physician** | | | |
| Heart failure | Asymptomatic with laboratory (e.g., BNP); cardiac imaging abnormalities | Symptoms with moderate activity or exertion | Symptoms at rest or with minimal activity or exertion; hospitalization; new onset of symptoms | Life-threatening consequences; urgent intervention indicated (e.g., continuous IV therapy or mechanical hemodynamic support) |
|  | *Demonstrated by at least one item in two of the following three categories*:*   - *Symptoms of heart failure (paroxysmal nocturnal dyspnea, shortness of breath, swelling, orthopnea, or weight gain)* - *Physical examination findings (presence of jugular venous distension, lung crackles, edema, or S3 heart sound)* - *Diagnostic testing (pulmonary edema on chest x-ray or brain natriuretic peptide >100 pg/mL, N-terminal pro-brain natriuretic peptide >125 pg/mL or Left Ventricular Ejection Fraction <50% or decreased over 10%* | | | |
| Angina (cardiac  chest pain) | Mild pain | Moderate pain; pain on exertion; limiting instrumental ADL; hemodynamically stable | Pain at rest; limiting self-care ADL; cardiac catheterization; new onset cardiac chest pain; unstable angina | - |
| Myocardial infarction | **-** | Asymptomatic and cardiac enzymes minimally abnormal and no evidence of ischemic ECG changes | Severe symptoms; cardiac enzymes abnormal; hemodynamically stable; ECG changes consistent with infarction | Life-threatening consequences; hemodynamically unstable |
|  | *Abnormal cardiac enzymes*:*   - *CKMB >6.6 ng/mL* - *Myoglobin >154.9 ng/mL (male) or 106.0 ng/mL (female)* - *Troponin I >0.028 ng/mL* | | | |
| Venous thromboembolism | Medical intervention not indicated (e.g., superficial thrombosis) | Medical intervention indicated | Urgent medical intervention indicated (e.g., pulmonary embolism or intracardiac thrombus) | Life-threatening consequences with hemodynamic or neurologic instability |
|  | *Evaluated by imaging** | | | |
| Pulmonary hypertension | Minimal dyspnea; findings on physical exam or other evaluation | Moderate dyspnea and cough; requiring evaluation by cardiac catheterization and medical intervention | Severe symptoms, associated with hypoxia and right heart failure; oxygen indicated | Life-threatening airway consequences; urgent intervention indicated (e.g., tracheotomy or intubation) |
|  | *Evaluated by echocardiography** | | | |

Table S2. Cumulative incidence of bortezomib-related cardiovascular adverse events

| **Cardiovascular adverse event** | **Number of events**  **(N)** | **6 months** | | **1year** | | **2year** | |
| --- | --- | --- | --- | --- | --- | --- | --- |
|  |  | **Cumulative incidence (%)** | **95% CI** | **Cumulative incidence (%)** | **95% CI** | **Cumulative incidence (%)** | **95% CI** |
| Any CVAE |  |  |  |  |  |  |  |
| Any grade | 82 | 16.90 | 12.91-20.90 | 26.38 | 20.82-31.95 | 32.09 | 24.79-39.40 |
| Severe | 58 | 12.04 | 8.60-15.47 | 17.99 | 13.30-22.68 | 21.65 | 15.94-27.36 |
| Hypertension |  |  |  |  |  |  |  |
| Any grade | 45 | 9.91 | 6.73-13.08 | 14.67 | 10.39-18.94 | 15.53 | 10.97-20.08 |
| Severe | 45 | 9.91 | 6.73-13.08 | 14.67 | 10.39-18.94 | 15.53 | 10.97-20.08 |
| Arrhythmia |  |  |  |  |  |  |  |
| Any grade | 10 | 1.61 | 0.33-2.90 | 3.88 | 1.33-6.44 | 3.88 | 1.33-6.44 |
| Severe | 2 | 0.25 | 0.00-0.75 | 0.75 | 0.00-1.85 | 0.75 | 0.00-1.85 |
| Heart failure |  |  |  |  |  |  |  |
| Any grade | 17 | 3.40 | 1.46-5.33 | 5.98 | 2.84-9.11 | 7.11 | 3.31-10.92 |
| Severe | 7 | 1.11 | 0.02-2.19 | 3.02 | 0.62-5.42 | 4.16 | 0.91-7.42 |
| Angina (cardiac chest pain) |  |  |  |  |  |  |  |
| Any grade | 13 | 2.37 | 0.70-4.04 | 4.39 | 1.59-7.19 | 13.13 | 0.00-28.10 |
| Severe | 3 | 0.29 | 0.00-0.84 | 0.29 | 0.00-0.84 | 9.34 | 0.00-24.74 |
| Myocardial infarction |  |  |  |  |  |  |  |
| Any grade | 5 | 1.04 | 0.03-2.05 | 1.04 | 0.03-2.05 | 4.20 | 0.00-10.38 |
| Severe | 2 | 0.27 | 0.00-0.81 | 0.27 | 0.00-0.81 | 3.46 | 0.00-9.63 |
| Pulmonary hypertension |  |  |  |  |  |  |  |
| Any grade | 11 | 2.10 | 0.53-3.67 | 3.86 | 1.34-6.38 | 4.74 | 1.71-7.77 |
| Severe | 2 | 0.00 | 0.00-0.00 | 0.70 | 0.00-2.07 | 1.59 | 0.00-3.78 |
| Venous thromboembolism |  |  |  |  |  |  |  |
| Any grade | 3 | 0.60 | 0.00-1.45 | 0.60 | 0.00-1.45 | 2.33 | 0.00-5.79 |
| Severe | 1 | 0.26 | 0.00-0.76 | 0.26 | 0.00-0.76 | 0.26 | 0.00-0.76 |
